# Basic reversal-learning capacity in flies suggests rudiments of complex cognition

**DOI:** 10.1371/journal.pone.0181749

**Published:** 2017-08-16

**Authors:** Brad R. Foley, Paul Marjoram, Sergey V. Nuzhdin

**Affiliations:** 1 Department of Molecular and Computational Biology, University of Southern California, Los Angeles, California, United States of America; 2 Department of Preventative Medicine, Keck School of Medicine of USC, Los Angeles, California, United States of America; Universitat Regensburg, GERMANY

## Abstract

The most basic models of learning are reinforcement learning models (for instance, classical and operant conditioning) that posit a constant learning rate; however many animals change their learning rates with experience. This process is sometimes studied by reversing an existing association between cues and rewards, and measuring the rate of relearning. Augmented reversal-learning, where learning rates increase with practice, can be an important component of behavioral flexibility; and may provide insight into higher cognition. Previous studies of reversal-learning in *Drosophila* have not measured learning rates, but have tended to focus on measuring gross deficits in reversal-learning, as the ratio of two timepoints. These studies have uncovered a diversity of mechanisms underlying reversal-learning, but natural genetic variation in this trait has yet to be assessed. We conducted a reversal-learning regime on a diverse panel of *Drosophila melanogaster* genotypes. We found highly significant genetic variation in their baseline ability to learn. We also found that they have a consistent, and strong (1.3×), increase in their learning speed with reversal. We found no evidence, however, that there was genetic variation in their ability to increase their learning rates with experience. This may suggest that *Drosophila* have a hitherto unrecognized ability to integrate acquired information, and improve their decision making; but that their mechanisms for doing so are under strong constraints.

## Introduction

The process of learning is a critical component of the way in which animals interact with the world, and respond to changing conditions. One of the most basic forms of learning is conditioned, or reinforcement, learning. In reinforcement learning, an animal learns that a conditioned stimulus (like a sound or odor) is predictive of an unconditioned stimulus (a reward, like sugar; or a punishment, like a bitter taste). The simplest neurons and ganglia are known to be capable of reinforcement [1, 2]. However, in many animals (whether bees, guppies, pigeons or mice [3–6]), when the association between conditioned and unconditioned stimuli is reversed, re-learning can take a different course than the initial conditioned response. This is called reversal-learning [7]. Reversal-learning is often more rapid than initial acquisition, and as such it is increasingly being studied as an important indicator of behavioral flexibility (e.g. [6, 8, 9]).

Because reversal-learning seems to be an ability to override simple conditioned associations, based on slower deliberation and higher order knowledge (such as an imputation of long-term consequences), reversal-learning is sometimes described as impulse control, or inhibitory control [10]. This simple description is being revised, however, given evidence that there are multiple modes of reversal-learning. For instance, pigeons seem to develop mental models, and make decisions based on their predictions of what state a system is in [5]. A distinction, then, is sometimes made between model-free (which does not depend on a mental “map” of endstates) versus model-based reversal-learning, which does integrate such a mental map [7, 11, 12]. A further distinction is sometimes made between model-free conditioned responses to cues, and highly conditioned, reflexive ‘habits’, which are stereotyped patterns of behaviors that may be insensitive to internal or external cues [13], which primarily result from operant conditioning.

We might postulate a general hierarchy of increasingly complex modes of learning—the simplest, conditioned, reinforcement learning; a second, more sophisticated, model-free reversal-learning; and a complex, model-based system of learning. In cognitively complex animals, reversal-learning results from a number of independent neural processes working in concert; including various neurotransmitters and neurotransmitter receptors, and specialised brain regions [7, 14]. Learning may proceed by conditioning to several cues simultaneously, at different rates—for instance, classical conditioning for visual cues can inhibit operant conditioning responses early in training, but be overridden by them, later in training, as they become ingrained habits [13]. The diversity of higher-order processes corresponds with variation in reversal-learning ability among individuals [10], and is known to have a genetic basis in some cases [15]. Inter-individual variation in reversal-learning ability in mammals has been shown to correlate with important behavioural traits like impulsivity, and propensity to addiction [6, 10]. It may also correlate with ecological function: in honey bees, for instance, variation in reversal-learning correlates with foraging roles, with the most exploratory scout caste showing the greatest capacity to reverse learn [3].

*Drosophila melanogaster* has long been a model of reversal-learning [16–20], and the outlines of the neurogenetics of reversal learning are known. There is evidence that the major contributor to reversal learning in the fly *Drosophila melanogaster* is the ability to actively suppress prior associations [16, 21, 22], and this suppression is controlled by the mushroom bodies, in conjunction with the anterior paired lateral neurons [9]. There may potentially be a minor role of adaptive forgetting in reversal learning [23], but this is less clear. Operant learning is known to proceed more slowly in *D. melanogaster* [20], to interfere with reversal-learning [13], and to have different genetic bases than classical conditioning [24, 25].

This relative complexity of the mechanisms underlying learning in *D. melanogaster*, suggests that there is a great deal of scope for inter-individual variation in cognitive flexibility. Until now, however, research has focused on genetic knockouts or chemical manipulations affecting gross memory phenotypes—reversal-learning has typically been measured as intensity of conditioning at two time-points; the “learning” time point, and the “reversed-learning” timepoint; and success of reversal-learning has been assessed as the relative degree to which the new association has been achieved. Natural genetic variation in reversal earning has not been assessed. Moreover, these experiments confound two different measures of learning. The degree of preference change has typically been used as a proxy for learning, however the *rate* of learning more accurately describes the speed at which associations are made (or remade). Here, we assess natural variation in the capacity to learn and relearn in *D. melanogaster*; and measure learning rate, by fitting reaction norms to preference across many timepoints.

In the Baseline Model section, we will introduce the classic conditioned-response model of learning. In the section on Evaluating Reversal Rate we will discuss how we can detect departures from this model, in the process of reversal-learning, by decomposing the learning rate into several independent components governed by genetics and experience. In the subsequent sections we describe an experiment we conducted, that allowed us to characterise these components in *D. melanogaster*.

## Baseline Model

When learning is simple reinforcement, it will resemble the pattern described by the Rescorla-Wagner model (cf [26, 27]). The Rescorla-Wagner (RW) model is the most commonly cited of the so-called “delta” models of reinforcement learning. Delta models describe the process of learning, or reinforcing, an association between an *unconditioned stimulus* (something that is *a priori* aversive, like an electrical shock; or pleasant, like food); and a *conditioned stimulus* (a cue, like a noise, or a flashing light, that is the target of the conditioning). In a delta model, with each paired exposure to the conditioned stimulus (or cue), and an unconditioned stimulus, the strength of the association between them changes. We will describe the value of the conditioned stimulus as *c*, and the value of the unconditioned stimulus as *u*. With each paired exposure, the strength of association, *w*, between the conditioned and unconditioned stimuli, is strengthened by an amount, Δ*w*. The value of the cue, *c*, is typically coded as a 1 or 0, denoting cue presence or absence. The change in the strength of the association, Δ*w* is proportional to the difference between the anticipated result, *a*, and the measured value, *u*. The greater this difference is, the more rapid the rate of conditioning:

$$\begin{array}{c} \Delta w=k(u-a)\cdot c \end{array}$$ (1)

Where *k* ℝ(0,1) is a learning rate term. The term *k* is a composite term, often written as *αβ* where *α* denotes the salience of the cue (some associations are easier to form than others)and the term *β* is a learning rate parameter, which may vary between individuals. The anticipated outcome, *a*, is simply the product of the cue presence or absence, and its weight:

$$\begin{array}{c} a=wc. \end{array}$$ (2)

The above learning process may be recursively iterated, such that strength of the association (or belief) at time *t*, *w*_*t*_, is the sum of the previous association, and the effect, Δ*w*_*t*−1_, of the training event at time *t* − 1:

$$\begin{array}{c} w_{t}=w_{t-1}+\Delta w_{t-1}. \end{array}$$ (3)

In order to calculate the association strength (belief) at any given time, *t*, for a constant conditioning regime, let us denote the initial belief as *a*_0_, and the empirical association between *u* and *c* as $\bar{u}$. Then, the belief at time *t* resulting from all prior learning events, *i* from time 0 to *t* − 1 is:

$$\begin{array}{c} w_{t}=w_{t-1}+\Delta w_{t}=a_{0}+\sum_{i=0}^{t-1}w_{i}+k\left(\bar{u}-w_{t-1}\right) \end{array}$$ (4)

We can solve Eq 4 for any time, *t*, as:

$$\begin{array}{c} w_{t}=\left(\bar{u}-a_{0}\right)\left[1-{\left(1-k\right)}^{t}\right]+a_{0}. \end{array}$$ (5)

Note that this equation contains a simple term, (1 − *k*)^*t*^, which describes the proportional change in the association strength, modified by several scale parameters, describing the initial difference in magnitude between the initial, and asymptotic, beliefs. This means, we can estimate the “half life” of learning, as a scale-free parameter. Importantly, whatever the error variance associated with *k* is, its contribution to (1 − *k*)^*t*^ will be asymmetric because of the exponent. Thus, our estimates of *k* will tend to be biased upward. For this reason, it is standard to evaluate such rate equations on a scale of log-time.

## Evaluating Reversal Rate

Given timecourse data from an iterated learning process as described above (Eq 5), we can estimate learning rate using 4 parameter sigmoid regression (see S1 Appendix). The midpoint parameter, $\hat{x}$, from the regression model is defined as the timepoint at which the strength of an association is halfway between the initial association and the final asymptotic association. The value of $\hat{x}$ is, however, independent of the values of the initial and final beliefs (i.e. it is scale invariant), and is a simple function of the underlying learning rate, *k* (S1 Appendix). Thus, $\hat{x}$ is a convenient metric of learning rate, and reversal-learning rate. If $\hat{x}$ decreases between the first learning experience, and the reversal period, it indicates that the learning rate has increased. If $\hat{x}$ increases between the first learning experience and reversal, it indicates that the first learned association is interfering with subsequent learning and that the learning rate is accordingly lower.

In order to investigate the processes underlying variation in learning, and to evaluate the components of learning using experimental data, we can decompose $\hat{x}$ to several components, related to prior learning and genetics, amenable to regression analysis:

$$\begin{array}{c} {\hat{x}}_{i,j}=L+G_{L,i}+R_{j}+G_{R,i,j}. \end{array}$$ (6)

We define *L* as the baseline learning rate, with an associated genotype-specific learning component, *G*_*L*,*i*_, for genotype *i*. The main effect of reversal, *j*, on learning rate is denoted by *R*_*j*_, and the associated genotype specific effect of reversal is described by *G*_*R*,*i*,*j*_.

In a regression framework, with terms for genotype and learning period, the overall learning rate, *L*, will be described by the intercept, and the genotypic learning rate, *G*_*L*,*i*_ will be described by the genotype term. The main reversal-learning effect, *R*_*j*_ will be described by the effect of learning period, and the genotype-specific reversal effect, *G*_*R*,*i*,*j*_ will be described by the interaction term between genotype and learning period.

## Materials and methods

In order to evaluate the above model, we took six genotypes of *D. melanogaster*, and measured their learning rates for developing an association between food odors (pineapple and grapefruit), and the presence or absence of a bitter compound (caffeine) in a binomial choice preference assay. We let them acclimatize to an arena, and then measured their food preferences as a function of time in a first “learning period”, and in a second “reversal-learning period”.

### Fly stocks

We used 6 genotypes. Each genotype was the F_1_ generation of a cross between two inbred strains. Six of the parental genotypes were inbred cosmopolitan lines from the Drosophila Genomes Reference Project (DGRP) Raleigh population [28]. Six of the parental genotypes were inbred Caribbean *D. melanogaster* [29]. We used these divergent populations to increase the chances that we would detect genetic variation in reversal-learning. Recurrent F_1_ flies from inbred lines derived from natural populations are a useful tool, because they are genetically identical, allowing for an estimation of genotypic variance, and represent a snapshot of naturally occurring genetic variation, while minimizing the problems associated with inbreeding depression and lab adaptation.

For each F_1_ genotype, one parental genotype was assigned as the paternal genotype, and a different one was the maternal genotype. Recurrent F_1_s were produced by rearing 5 paternal genotype males, and 20 maternal genotype virgin females in standard food vials, and collecting the offspring. F_1_, experimental, flies were collected on the first day post eclosion, and reared in mixed sex, density controlled, full sib, vials for 3 more days, to ensure all females were mated. Flies were sexed under minimal CO_2_ anaesthesia, lasting no more than 5 minutes, males were discarded, and females were allowed to recover for at least 24 hours before being tested. We tested females who were between 4 and 8 days old. While strong and prolonged CO_2_ anaesthesia has been shown to lead to certain behavioural deficits [30, 31], minimal anaesthesia has not.

We tested mated females for several reasons. Females eat much more than males [32], and are thus more likely to repeatedly experience the association between bitterness and food odour. Females are also less territorial, and more commonly find themselves in large aggregations, enabling us to test learning in bulk. Finally, we have evidence that males are generally more likely to move more frequently between food patches, perhaps limiting our ability to detect preferences [33].

### Food

The food was made of agar (2%) and yeast (3.5%); with deionized water and enough juice (either pink grapefruit or pineapple) to bring the total sugar content to 5.9% (approximately half the medium, by volume). The two juices were chosen to provide different, but equally attractive, olfactory cues. Previous tests have shown that both pineapple and grapefruit juice are highly attractive to *D. melanogaster*, and preliminary trials showed that naïve preferences for the two foods was indistinguishable. For the bitter (aversive) food, we took the basic medium (attractive food) and brought it to a concentration of 0.4% (approximately 20mM) caffeine. This concentration is more than adequate to elicit a highly aversive response [34]. The food mixture contained half the total sugar content of our normal fly preference assays [33], and no alcohol, because sugar and alcohol were found to hinder discrimination of caffeine.

### Experimental setup

**Fig 1:**
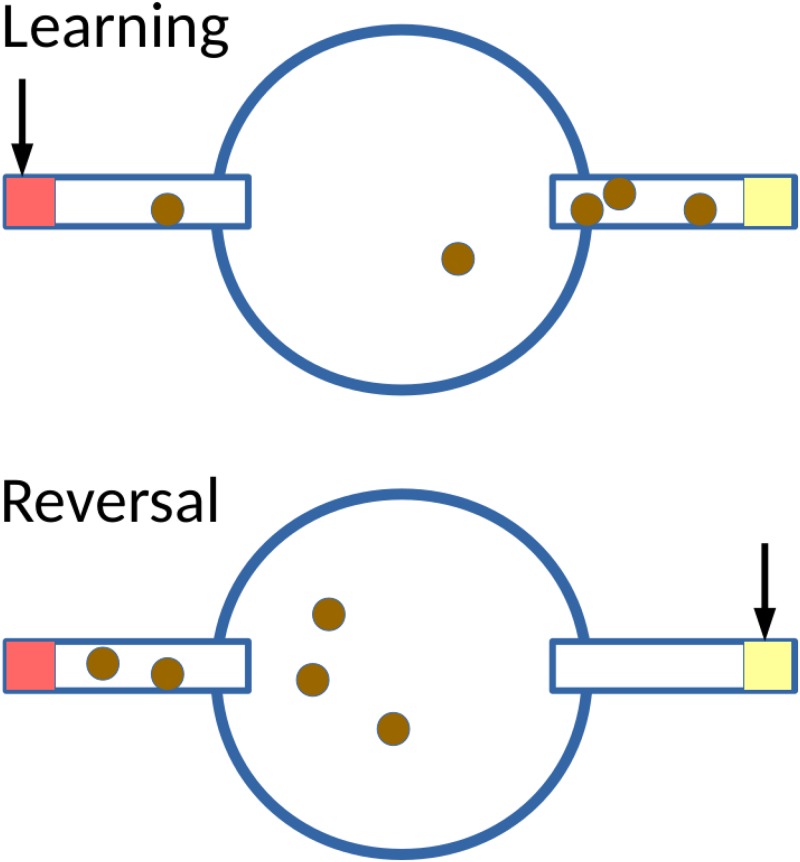
The experimental setup for this experiment. A central chamber leads into two separate food sources, either pineapple or grapefruit. In the learning period, following 8 hours of acclimation, one is made bitter (indicated with the arrow). In the reversal-learning period, the bitterness of the foods is reversed. Flies (dark dots) are allowed to move between the food sources, and their preferences across time are recorded.

The physical arena consisted of a 2cm diameter, 2cm height, central chamber composed of transparent polystyrene (constructed from laboratory fly rearing vials), sealed from the top with a foam stopper (Fig 1). Two holes were made in the inner chamber, opposite each other. These holes led to small food chambers. The food chambers were made of 5cm lengths of transparent drinking straws. The distal end of these straws contained a 0.5cm plug of food, and was sealed. Each setup had one grapefruit food option, and one pineapple food option, to allow for olfactory discrimination. Flies could move freely around the chambers. Parafilm was used around the base of each food chamber to ensure a snug fit between it and the central chamber.

## Results

Overall, in both the training and reversal periods, flies initially investigated both foods, but quickly learned to avoid the bitter (aversive) food and developed a preference for the non-aversive food. We investigated food odor (grapefruit or pineapple) as a covariate in all the following models, but it was never strongly significant and we will not report on it further. We will, instead, simply describe patterns of attraction to the aversive vs non-aversive associated stimulus.

### Time course of choice

**Fig 2:**
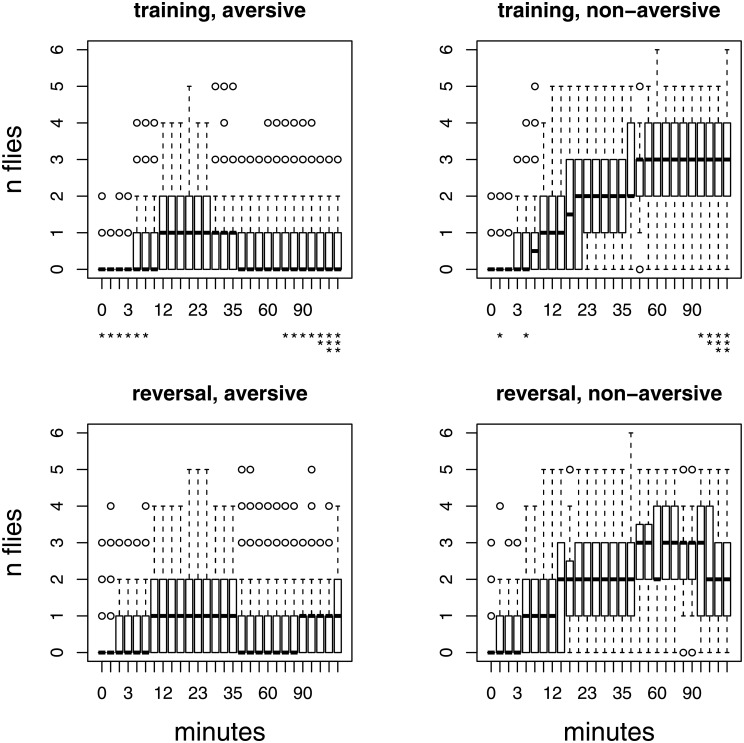
Box plots of fly count on the aversive and non-aversive food patches, by measurement time (in minutes). All trials, with five flies each, for each time period are shown together. The top row shows the first, training, period (n trials = 118). The bottom row shows the reversal period (n trials = 123). The timepoints where the initial and reversal periods are significantly different for presence on patches are indicated by asterisks (* < 0.05, ** < 0.01, *** < 0.001). P-values were not corrected for multiple testing. Data points more than 1.5 times the interquartile range away from the mean are indicated by open circles.

Before testing learning rate, using the fitted model of Equation 1 in S1 Appendix, we tested each time-point individually. We wished to evaluate whether we could detect differences between learning and reversal learning; and whether there were effects of genotype and day. We conducted the analysis across all trials, and all genotypes. Each time point represents 118 to 123 trials, of 5 flies each. In both periods, the pattern is similar. Flies initially explore the aversive and non-aversive options, and quickly learn to avoid aversive food (Fig 2).

In order to evaluate the significance of the effects of period, genotype, and day, on food choice at each time point, we conducted conducted multiple linear regression. We tested for the effect of period (either learning period, or reversal-learning period) on the number of flies on each kind of food at each time point, for aversive and non-aversive food separately. We used a multiple regression model (glm{stats}) in R, including genotype and day effects as covariates, modelled as factor variables, with marginal significance for each term estimated using Type II ANOVA (Anova{car}), with orthogonal contrasts appropriate for unordered factors, and a Poisson distribution. In all cases, the tests had 243 residual degrees of freedom. The effect of period was most evident for time points at the start and end of the two hour intervals. Flies were quick to move to the aversive food in the reversal-learning trial. For minutes 0 through 7 there were more flies on the aversive food in the reversal-learning period than in the initial learning period. But, after exploring their previously preferred food, fies switched more quickly to the non-aversive food in the reversal-learning period than in the learning period. Between minutes 1 through 10, flies were consistently, but weakly, more likely to be on the non-aversive food in the reversal-learning-period. There was also a difference between the two periods towards the end of the time course. Between minutes 75 and 120, fly preferences for non-aversive food, and aversion to bitter food, became weaker—but only in the reversal period.

We did not correct for multiple testing, since our aim here was a general description of the timecourse; however, the number of significant tests for the effect of period greatly exceeds the random expectation, given 28 tests. There were significant preference differences between the learning and reversal learning periods at 12 of the 28 timepoints for the aversive food; and 6 out of 28 timepoints for the non-aversive food (by chance, we would only expect 0.05 × 28 = 1.4 tests significant at the *p* < 0.05 level.) Genotypes differed in their overall preferences as well. Looking at partial F-tests, genotype was significant in 17 of the 28 tests on non-aversive food, and in 22 of the 28 tests on non-aversive food. Similarly, there were strong day effects. Day was significant in 17 of the tests, for aversive food; and in 15 of the tests for non-aversive food.

**Fig 3:**
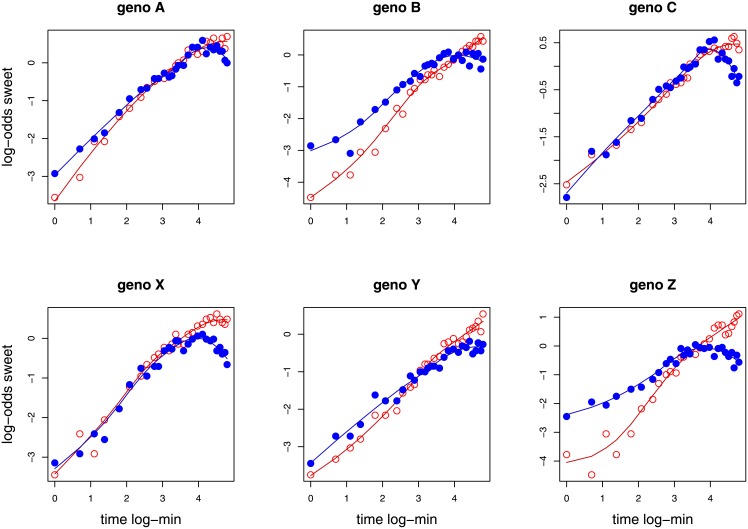
Log-preference ratios for the non-aversive (sweet) food across time (in log minutes), for each genotype. All observations for each time period for each time period, learning and reversal-learning, were pooled, and preferences estimated. Thus, each point represents the choices of 85 to 119 individual flies. Open (red) points indicate the initial learning period; filled (blue) points indicate observations in the reversal-learning period. Lines are fitted with loess{R} regression.

Learned preference for the non-aversive food was a good qualitative fit to the anticipated sigmoid curve, for all genotypes, in the learning and reversal-learning periods (Fig 3). Note that too few flies were present on the non-aversive food at any time point through the course of the experiment to fit meaningful learning curves. For some genotypes, there was a slight weakening of preference in the last few observations of the reversal-learning period.

### Reversal-learning

**Fig 4:**
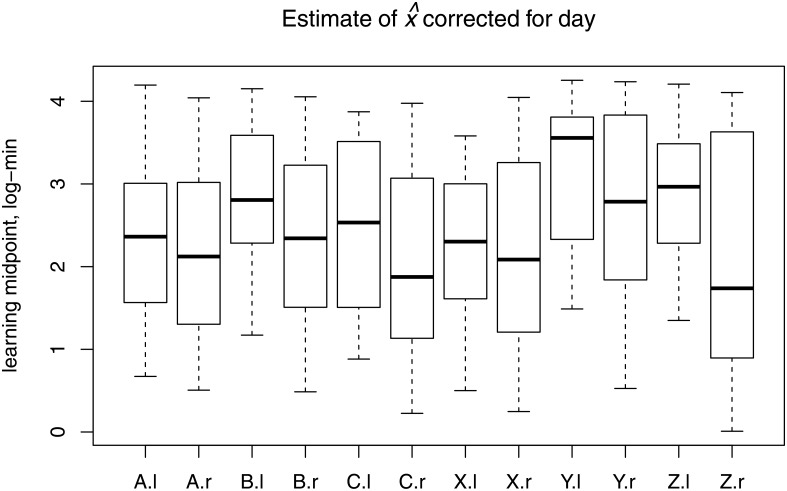
Box-plots showing the estimated midpoints, $\hat{x}$, of the learning curves for all 6 genotypes, in the learning (l) and reversal (r) periods. Learning rates have been corrected for day effects, for maximum clarity of presentation, uncorrected results are shown in S2 Fig.

For each time period, for each trial (containing 5 flies), we estimated the midpoint of the learning curve $\hat{x}$—that is, a proxy for learning rate (Fig 4). There were evident differences among genotypes, and for each genotype, $\hat{x}$ is lower in the reversal period than in the learning period.

We fitted the model of Eq 6 using multiple regression, to evaluate the significance of the terms contributing to learning rate. The full model included genotype, a variable indicating whether data are from the initial learning (period = 0) or reversal-learning (period = 1) periods, and genotype×period terms. We used the midpoint estimates, $\hat{x}$, of learning rate as the response variable. The intercept term of the regression model, *α*_*L*_, corresponds to *L*—the baseline learning rate (Eq 6). The genotype-specific learning rates, *G*_*L*,*i*_ correspond with the genotypic estimates associated with the genotype regression term *β*_*G*_; the overall effect of reversal on learning rates, *R*_*j*_, corresponds with the period regression coefficient, *β*_*R*_, and the genotype-specific reversal component, *G*_*R*,*i*,*j*_, corresponds with the regression coefficient on the genotype×period interaction term, *β*_*G*×*R*_. We used multiple regression (glm{stats}) in R, as above, and included date as a block-effect covariate.

The marginal p-value (type III ANOVA) of the genotype×period interaction, *β*_*G*×*R*_, was not significant (*β*_*G*×*R*_: *χ*^2^ = 4.48, *df* = 5, *p* = 0.48), thus we did not include the interaction term in the preferred model. *β*_*G*×*R*_ never approached significance, regardless of the smoothing parameter that was used. The preferred model included genotype and period. Considering the marginal significance of all terms (type II ANOVA), the effect of period, (*β*_*R*_: *est* = −0.35, *χ*^2^ = 7.82, *df* = 1, *p* = 0.005), genotype (*βG*: *χ*^2^ = 20.7, *df* = 5, *p* < 0.001), and block effect (*χ*^2^ = 23.2, *df* = 6,*p* < 0.001) were all significant.

The estimated effect of reversal on learning rate was *e*^−0.35^ = 0.70. That is, the average midpoint estimate in the reversal-learning period was 10.7 minutes; which is approximately 30% faster than the midpoint estimate of 15 minutes in the initial learning period. The correlation in genotypic means between the learning and reversal-learning periods was high, with a Pearson correlation of 0.64.

We validated model goodness-of-fit using permutation. We permuted learning rates with respect to genotype for 1 × 10*e*5 iterations. For each iteration we performed multiple regression, exactly as above, with date, period, and genotype effects. We recorded the proportion of deviance explained by the model, and found that deviance explained by the real data was higher than chance, in all but one permutation (i.e. p = 0.00001). We also explored the effects of keeping the period associations within day intact, in case replicate effects were biasing our estimates, and found that the deviance explained by the real data was still higher than chance, with p = 0.0001.

## Discussion

*Drosophila melanogaster* demonstrates an ability to learn and make choices that is consistent with some kind of reversal-learning effect, but inconsistent with the model of simple conditioned response learning (Eq 5). In our experiment, flies first learned to associate food odors with a bitter stimulus, and then reversed this preference more quickly than they formed the initial association. In a strict RW framework, the reversed associations should be gained at the same rate as the initial associations, but we have shown that learning rate increases with experience. At the very least, this implies some kind of higher-order integration of beliefs about the world, perhaps consistent with so-called “model-free” reversal-learning [7, 11]. Flies all show a consistent proportional change in their learning rates with experience, however, indicating some kind of constraint on this reversal-learning capacity.

When initially presented (in the learning-period) with paired bitter and non-bitter food, *D. melanogaster* was found on both foods with similar frequency, and only started to show marked preference for the non-bitter food after about 10 minutes (Fig 2). In the subsequent, reversal-learning period, flies showed an immediate preference for the previously non-bitter food—demonstrating that they have, in fact, made an association between fruit odor and the food quality. This initial preference quickly began to reverse, and within about eight minutes, flies showed a marked preference for the non-aversive food. While reversal-learning ability is well known in *D. melanogaster* (e.g. [17, 19]), an augmented learning rate with experience has not been demonstrated before. This pattern of learning to learn, however, is generally consistent with results in more complex animals such as bees [3].

We see the same general pattern when we examine the effect of period on the learning midpoint, $\hat{x}$. Flies in the learning-period take approximately 1.3× the amount of time to reach the midpoint of their maximal association between bitterness and food odor, as in the reversal-learning-period. This corresponds with a change from 15 minutes, to 10.7 minutes with increased experience. Accordingly, the regression component for period, *β*_*R*_, was highly significant. This kind of accelerated learning with experience has not been noted in *Drosophila* before. But the uniqueness of this observation may be more apparent than real, because learning rates *per se* have not often been assessed. For invertebrates, learning and reversal learning is typically described in terms of magnitude metrics, not rates. Apart from work on bees [35], few studies of learning explicitly evaluate learning midpoints—even when the data collected is amenable to this analysis. A large number of studies of learning focus on single-step saturation training [20]; for instance [17, 18, 36]. Reversal learning evaluation is typically the comparison of single-estimate post-training, and post-reversal levels of conditioning (including [8, 21]). In other experiments, time course data is aggregated, and these single metrics are compared [13, 25].

An important exception to this is the experimental paradigm of Ren *et al* ([9]), which assays changes in preference at several distinct timepoints; and found an apparent deficit in learning rate on reversal—although this did not account for different baseline (initial) beliefs. The design of Ren *et al* consisted of a single round of constant training within a 30 minute block, and reversal at 15 mins. Thus one important difference between the two experiments may involve the relative importance of long-term versus short term memory. *Drosophila* learning is known to involve three distinct phases [37, 38]. Short term (sometimes called Anaesthesia Sensitive Memory, or ASM) operates on scales of minutes) and is distinct from medium term memory (Anaesthesia Resisant Memory, ARM) which operates on scales of several hours, and these are distinct again from Long Term Memory (LTM, often assayed after a gap of 24 hours post-conditioning). The different phases of memory may have differential susceptibility to being overridden.

The current experiment and that of Ren *et al* ([9]) differed, as well, in the stimuli being presented and the responses being assayed. In Ren *et al*, flies in flight were punished, with heat, for turning the wrong direction when presented with a visual cue. In our experiment, the cues were mainly olfactory and gustatory. Flies may be predisposed to learn to evaluate associations between bitter and olfactory cues rapidly.

We also found that there was a great deal of variation among genotypes in their learning rates, reflected in the significance of the genotype regression coefficient, *β*_*G*_. This is consistent with other experiments that have found genetic variation in fly learning [39, 40]. However, despite differences in their baseline ability to learn, and despite flies’ general ability to learn more quickly in the reversal period, there was no indication that genotypes varied in their ability to increase their learning rates in the reversal-learning period. That is, the genotype-specific reversal-learning interaction term, *β*_*G*×*R*_, in the regression model was non-significant, and across genotypes learning and reversal-learning rates were highly correlated (0.64).

This result—a lack of genotypic variation for processes specific to reversal-learning in *D. melanogaster*—is something of a surprise, given the complexity of the phenotype. Mutant screenings have found multiple genes causing different deficits that uncouple reversal-learning from initial learning [8, 16, 19, 20]. There are also neural mechanisms specific to reversal learning, in particular mushroom body neurons that suppress old memories [9, 13, 21]. With such a broad target for gene-expression, and developmental variation, we might expect to see genotypic differences in components of reversal learning. It is unclear what evolutionary or developmental constraints might restrict the expression of variation in this trait.

The increase in flies’ ability to choose suitable foods, given prior exposure to unsuitable options, suggests that they are integrating more complex information in their learning and decision making, in the process of reverse learning. The precise form this integration might take in *Drosophila* is unclear. The simplicty and robustness of the flies’ response to reversal, however, suggest that *Drosophila* may be a suitable model for studying these most basic forms of higher cognition.

## Supporting information

S1 Appendix Extended mathematical and experimental methods. Detailed description of sigmoid regression, midpoint estimation, and experimental design and cross details.
(PDF)

S1 Fig A random sample of 8 trials, out of a total of 241, showing estimation of learning responses. Log-ratio preference scores are shown (black dots), on a scale of log-minutes. The smoothed learning curve, fitted with loess regression, and span-parameter 1.45 is shown by the dotted line; and the estimated learning midpoints (red dots). Each figure presents a single learning or reversal learning period, for a single time period, for a single trial; thus each dot is the summed behavior of five flies of a single genotype.
(EPS)

S2 FigBox-plots showing the estimated midpoints, $\hat{x}$, of the learning curves for all 6 genotypes, in the learning (l) and reversal (r) periods.Learning rates have not been corrected for day effects. Corrected results are shown in Fig 4.(EPS)

S3 Fig The experimental design of the current experiment. Showing, the construction of the F_1_ genotypes from inbred lines, the composition of each trial, and the 3 stages of each trial; as well as the number of replicate individuals within trials, trials within genotype; and total trials conducted.
(EPS)
