# Supplementary material for: Basic reversal-learning capacity in flies suggests rudiments of complex cognition
Source: PLoS One. 2017 Aug 16;12(8):e0181749. doi: 10.1371/journal.pone.0181749 (PMC5558953; doi:10.1371/journal.pone.0181749)
Supplement: S1 Appendix — Detailed description of sigmoid regression, midpoint estimation, and experimental design and cross details. (PDF) [file pone.0181749.s001.pdf]

Appendix

Sigmoid regression and learning rate estimation

We can fit curves resulting from iterated exponential processes (like conditioned learning under Rescorla-Wagner), using nonlinear regression for a four-factor sigmoidal curve, on a scale of log-time, $x = log(t+1)$. The logic for using a scale of log-time is outlined in the section "Baseline model". This curve is defined by the lower asymptote, a_0, and the upper asymptote, $\bar{u}$; as well as a rate term, b, and the midpoint of the curve—$\hat{x}$.

$$w_x = a_0 + (\bar{u} - a_0)/(1 + e^{b(\hat{x}-x)}) \quad (1)$$

One way to estimate learning rate is to calculate the midpoint of the learning curve, Eq 4. To capture the departure of reversal-learning from the baseline RW model, we can estimate the change in $\hat{x}$ in subsequent, reversed, association tasks. If $\hat{x}$ does not change significantly from one reversal to another, the underlying model of learning cannot be distinguished from the basic RW model. If $\hat{x}$ increases between learning events, we may infer that the learning rate has decreased, and there is some kind of interference between earlier and later associations. If $\hat{x}$ decreases between learning events, we may infer that the learning rate has increased; that is, that animals are learning to be better learners.

For simplicity, we will denote the difference between the initial belief, a_0, and the maximum belief $\bar{u}$, as $\lambda = \bar{u} - a_0$. The midpoint occurs where $w_x = \lambda/2$. Solving for x:

$$\lambda/2 = \lambda[1 - (1-k)^x] \quad (2)$$

$$x = \frac{log(1/2)}{log(1-k)} \quad (3)$$

Note that the term λ cancels out, thus the midpoint is independent of the difference between the lower and upper asymptotes; and depends solely on k, i.e. the salience, and associability of the cue. This property makes the midpoint a very convenient metric of learning rate.

If we solve for k, to try and understand the relationship between learning rate and midpoint, we see:

$$k = 1 - e^{-0.693/log(t+1)} \quad (4)$$

Midpoint estimation

The midpoints were estimated as the halfway point between the first local minimum and the global maximum, using the loess curve (Fig A1). Visual inspection showed that these estimates fell out very well, relative to the distribution of the data. We examined all replicates for outliers, and here show 8 graphs, drawn at random from all the time periods and all the trials, for illustration. We estimated 241 learning midpoints, in total, and we show 8 randomly selected trials for illustrative purposes. In all cases, the points fall in the bounds of the graph, in a region where fly preference is rapidly moving to the maximum. These heuristic estimates were far more robust than maximum likelihood estimates, obtained using nonlinear regression. Attempts to estimate midpoints using sigmoid regression (for instance, using the package nplr) were fragile, with high rates of

S1 Fig. A random sample of 8 trials, out of a total of 241, showing estimation of learning responses. Log-ratio preference scores are shown (black dots), on a scale of log-minutes. The smoothed learning curve, fitted with loess regression, and span-parameter 1.45 is shown by the dotted line; and the estimated learning midpoints (red dots). Each figure presents a single learning or reversal learning period, for a single time period, for a single trial; thus each dot is the summed behavior of five flies of a single genotype.

non-convergence, even on aggregated data, and unreasonable estimates, between -5 and 45 logged-minutes.

Across all trials, within each genotype, the estimated value of $\hat{x}$, in log-minutes demonstrate a great deal of inter-genomic variation (Fig. A2), as well as distinctly lower reversal learning rates. Analysis of the significance of genotype, period and day effects are presented in the results.

S2 Fig. Box-plots showing the estimated midpoints, $\hat{x}$, of the learning curves for all 6 genotypes, in the learning (l) and reversal (r) periods. Learning rates have not been corrected for day effects. Corrected results are shown in Fig 4.

Experimental design, and cross information

The Cosmopolitan recurrent F_1 genotypes that we used (here designated A, B, and C) are genotypes we have used previously [1]. They were constructed as the following maternal×paternal crosses (where numbers are the genotype accession numbers for the Drosophila Genome Reference Project) correspond with accession numbers RAL-360×RAL-335, RAL-732×RAL-774, RAL-486×RAL-380. The Caribbean genotypes (here designated X, Y, Z) were similarly constructed by crosses between inbred lines collected in the Caribbean [2], kindly provided by R. Yukilevich.

S3 Fig. The experimental design of the current experiment. Showing, the construction of the F_1 genotypes from inbred lines, the composition of each trial, and the 3 stages of each trial; as well as the number of replicate individuals within trials, trials within genotype; and total trials conducted.

References

1. Saltz JB. Natural genetic variation in social environment choice: context-dependent gene-environment correlation in *Drosophila melanogaster*. Evolution. 2011;65(2325-2334).

2. Yukilevich R, True JR. Incipient sexual isolation among cosmopolitan *Drosophila melanogaster* populations. Evolution; international journal of organic evolution. 2008;62(8):2112–21.
